# Development and Characterization of In Situ Gelling Nasal Cilostazol Spanlastics

**DOI:** 10.3390/gels11020082

**Published:** 2025-01-22

**Authors:** Maryana Salamah, Mária Budai-Szűcs, Bence Sipos, Balázs Volk, Gábor Katona, György Tibor Balogh, Ildikó Csóka

**Affiliations:** 1 Institute of Pharmaceutical Technology and Regulatory Affairs, Faculty of Pharmacy, University of Szeged, Eötvös Str. 6, H-6720 Szeged, Hungary; salamah.maryana@szte.hu (M.S.); budai-szucs.maria@szte.hu (M.B.-S.); sipos.bence@szte.hu (B.S.); csoka.ildiko@szte.hu (I.C.); 2 Institute of Pharmacodynamics and Biopharmacy, Faculty of Pharmacy, University of Szeged, Eötvös Str. 6, H-6720 Szeged, Hungary; 3 Directorate of Drug Substance Development, Egis Pharmaceuticals Plc., Keresztúri Str. 30–38, H-1106 Budapest, Hungary; volk.balazs@egis.hu; 4 Department of Pharmaceutical Chemistry, Semmelweis University, Hőgyes Endre Str. 9, H-1092 Budapest, Hungary; 5 Center for Pharmacology and Drug Research & Development, Semmelweis University, Üllői Str. 26, H-1085 Budapest, Hungary

**Keywords:** cilostazol, spanlastics, in situ gel, Phytagel^®^, Poloxamer-407, chitosan

## Abstract

Cilostazol (CIL), a BCS class II antiplatelet aggregation and vasodilator agent, is used for cerebrovascular diseases to minimize blood–brain barrier dysfunction, white matter-lesion formation, and motor deficits. The current work aimed to develop and optimize cilostazol-loaded spanlastics (CIL-SPA) for nose-to-brain delivery to overcome the low solubility and absorption, the first pass-metabolism, and the adverse effects. The optimal CIL-SPA formulation was loaded into Phytagel^®^ (SPA-PG), Poloxamer-407 (SPA-P407), and chitosan (SPA-CS) gel bases and characterized in terms of colloidal properties, encapsulation efficiency (EE%), mucoadhesive properties, and biopharmaceutical aspects. The developed in situ gelling formulations showed a <300 nm average hydrodynamic diameter, <0.5 polydispersity index, and >|±30| mV zeta potential with a high EE% (>99%). All formulations met the droplet size-distribution criteria of nasal requirements (<200 µm), and all formulations showed adequate mucoadhesion properties. Both the BBB-PAMPA and horizontal permeability study through an artificial membrane revealed that all formulations had higher CIL flux and cumulative permeability at in vitro nose-to-brain conditions compared to the initial CIL. The in vitro drug-release study showed that all formulations released ca. 100% of CIL after 2 h. Therefore, the developed formulations could be promising for improving the low bioavailability of CIL through nose-to-brain delivery.

## 1. Introduction

Cerebrovascular diseases (including ischemic stroke, intracerebral bleeding, vascular cognitive impairment, and chronic cerebral hypoperfusion) are the most serious neurological disorders, representing the second leading cause of mortality in the world, the fifth cause of death in the US, and one of the top 10 causes of childhood death [1,2,3,4].

Treatment of central nervous system (CNS) diseases has many challenges, including physiological barriers that limit the transportation of drugs, such as the blood–brain barrier (BBB), blood–cerebrospinal fluid barrier (BCSFB) [5,6], and the rapid elimination from the brain and cerebrospinal fluid (CSF) [7].

Intranasal administration offers an effective route for nose-to-brain delivery (through the olfactory and trigeminal nerves) to overcome the BBB and BCSFB [8,9], improve the absorption due to the large nasal mucosa surface area, and reduce the adverse effects [7,10]. However, intranasal administration has some obstacles, including rapid mucociliary clearance, which decreases the residence time of the administered formulation (clearance half-life of about 15 min) [11], and the particle size, which affects drug permeability through the nasal mucosa [5,12].

For intranasal administration, the drug-delivery carrier is a critical parameter for the absorption and penetration of the drug through the nasal epithelium [13]. Nanovesicular delivery systems, such as liposomes and niosomes, have been widely used to encapsulate hydrophilic and hydrophobic drugs [14,15]. Spanlastics are sorbitan-based nanovesicular systems which are modified from niosomes by using edge activators instead of the cholesterol, thus adding flexibility and elasticity to the vesicle membrane [16]. The incorporation of penetration enhancers within the surfactant bilayers improves the fluidization of the spanlastic vesicles and the penetration through the nasal epithelium and BBB [17,18].

Cilostazol (CIL) is an antiplatelet drug with vasodilation properties [19,20]. It was approved by the United States Food and Drug Administration (US FDA) and the European Medicines Agency (EMA) for the treatment of ischemic symptoms and intermittent claudication [21]. CIL has been reported to be neuroprotective against cognitive impairment and apoptotic white matter damage in rats [22,23] and to promote neuronal repair following the cerebral ischemia [24]. CIL belongs to the Biopharmaceutics Classification System (BCS) class II drugs (low water solubility, <6 µg/mL at 37 °C, and high permeability) [25,26] and has a moderate lipophilicity (log P = 2.72) [27]. Common adverse effects of CIL are related to its vasodilatory and inotropic properties, including headache, tachycardia, and palpitation [21].

In situ gelling formulations can be stored initially in a liquid state, but after delivery to the nasal cavity, sol-gel transition occurred due to hydrophobic interactions of the gel components, triggered by various physical stimuli (temperature, pH, and ionic concentration of the nasal mucosal environment) or chemical interaction (oxidative cross-linking). The incorporation of spanlastic colloidal dispersions into an in situ gel system (thermosensitive, pH-sensitive, and cation-sensitive gel) has been proposed as an alternative carrier to enhance the stability, prolonging drug residence time in the nasal cavity and promoting the release rate and the absorption [28,29,30,31].

We envisaged that intranasal administration of CIL could contribute to improved targeted drug delivery to the brain, therefore reducing side effects. The present study aims to prepare, characterize, and compare cilostazol-loaded spanlastic vesicles (CIL-SPA) and their incorporated form into various in situ gelling matrices intended for nose-to-brain delivery. In situ gelling nasal formulations are expected to overcome the low oral-bioavailability of CIL by improving the solubility and extending the residence time on the nasal mucosa, which improves the absorption and permeability through the nasal barrier.

## 2. Results and Discussion

### 2.1. Formulation of Cilostazol-Loaded Spanlastic Formulations (CIL-SPAs)

The CIL-SPAs were successfully prepared by the ethanol (EtOH) injection method. It is the most commonly used method because of its simple and reproducible technique [32,33,34]. In this study, we used Span^®^ 60 as a nonionic surfactant for the fabrication of spanlastics due to its hydrophilic–lipophilic balance (HLB) value (4.7), which is within the optimal HLB range (4–8) for formation in the vesicles [16,35]. Furthermore, the lipophilic nature of Span^®^ 60 (long chain length (C18)) leads to a higher encapsulation efficiency compared to other Span surfactants, it has high stability, and it is less disturbed and aggregated [17,36,37]. The other component used to fabricate spanlastics in this study is Tween^®^ 80 (HLB 14.9) as a nonionic edge activator, which gathers at the location of enhanced pressure at a specific site to reduce interfacial tension and free energy for shape modification to facilitate flexibility; thus, spanlastics can easily squeeze through the narrow pores (penetration enhancer) [38,39,40]. Tween^®^ 80 effectively formed smaller, nanosized vesicles [41], which showed more elasticity and deformability than Tween^®^ 40 and 20 due to the flexible and short polyoxyethylene polymer chains of Tween^®^ 80 and the existence of an unsaturated alkyl chain (double bond), as reported by Badria and Mazyed (2020) [42]. Their study showed that the optimal Span^®^ 60: Tween^®^ 80 ratio was 80:20 (using 400 mg Span^®^ 60 and 100 mg Tween^®^ 80), which conforms to other studies [16,43,44,45]. Furthermore, we used oleic acid as a penetration enhancer, in addition to its role as an edge activator. It is a naturally occurring fatty acid widely used in nanoparticles to increase stability and cellular uptake [46]. Different types of penetration enhancers were used in previous studies to prepare spanlastics with a concentration of 1% *w*/*v*; thus, we also selected this ratio in our study [43,44,47]. All materials used to prepare spanlastics are approved by US FDA and are generally recognized as safe (GRAS) [48].

After injecting the ethanolic solution into an aqueous solution of Tween^®^ 80, the resulting spanlastic dispersion was stirred at 60 °C to ensure the evaporation of EtOH, followed by sonication at 80% amplitude. Mary et al. (2022) studied the effect of the intensity of the sonication ion on the spanlastic vesicles and observed that an amplitude of 80% resulted in high cavitation intensities within the liquid phase and thus produced smaller vesicles [49]. Badria and Mazyed (2020) [42] and Zaki et. al. (2022) [44] studied the impact of sonication time on vesicle size and encapsulation efficiency (EE%). Their studies indicated that increasing the sonication time to 5 min decreased the size of the vesicles and EE% due to drug leakage to the external aqueous environment containing a surfactant, and the vesicles remained in the aqueous phase due to micellar solubilization instead of being entrapped within the vesicles. For these reasons, we fixed the sonication amplitude at 80% for 2 min.

### 2.2. Formulation of Cilostazol Spanlastic-Loaded In Situ Gel

In this study, we used various gel bases, such as Phytagel^®^, Poloxamer-407, and chitosan, to prepare CIL spanlastic-loaded in situ gelling formulations. Phytagel^®^, known as gellan gum, is a natural anionic heteropolysaccharide which exhibits mucoadhesive properties. It has been reported as biocompatible for pharmaceutical and human use (approved by the US FDA) [50,51,52]. It is soluble in water and can form an in situ gel in the presence of cations, which exist in the nasal fluid, due to physical cross-linking by covalent or non-covalent bonds [52,53]. It has been used in nasal drug delivery systems for several drugs (gastrodin [54], metoclopramide [55], fluoxetine [56], donepezil [57]).

Poloxamer-407, also known as Pluronic F127^®^, is a tri-block copolymer with amphiphilic properties in aqueous solutions; thus, it self-assembles into micelles, the core of which consists of a hydrophobic polypropylene oxide chain and the shell of two hydrophilic polyethylene oxide chains [58,59]. Poloxamer-407 is a biocompatible polymer and approved by the US FDA for pharmaceutical applications [60]. It undergoes a phase transition near body temperature, resulting in the formation of a thermo-responsive in situ gel [61,62]. It has been used in nasal delivery systems for several drugs due to its gelling behavior, mucoadhesive properties, and biocompatibility (dolutegravir [63], quercetin [64], zolmitriptan [65]).

Chitosan is a natural cationic linear polysaccharide, biocompatible, with low toxicity, and it can be degraded by human enzymes [66]. It is approved as GRAS by the US FDA [67]. Chitosan is water-soluble under acidic conditions, and through the impact of physical/chemical parameters (temperature, pH, and ionic strength), it can form a physical in situ gel due to electrostatic and hydrophobic interactions, hydrogen bonds, or ionic interactions (in a typical pH range of 4 to 6) between its positive charge and negatively charged ions, such as sialic acid in the nasal mucosa [68,69]. Thus, chitosan gel is considered a thermosensitive, pH-sensitive, and cation-sensitive gel [70]. It has been used in nasal delivery systems for several drugs (telmisartan [71], imipramine [72], propranolol [61]).

The final concentration of each polymer was determined based on a preliminary study by checking the visual viscosity of each gel base in three different concentrations: 0.1, 0.2, 0.3% *w*/*v* for Phytagel^®^ (SPA-PG), 10, 12, 14% *w*/*v* for Poloxamer-407 (SPA-P407), and 0.5, 1, 2% *w*/*v* for chitosan (SPA-CS) using Simulated Nasal Electrolyte Solution (SNES) 1:1 *v*/*v* at 32 °C.

### 2.3. Fourier Transform Infrared Spectroscopy (FTIR) Analyisis

The FTIR measurement was used to understand the interaction (peak shifts or changes) between the drug and excipients (Figures S1–S4). The FTIR spectra of CIL showed N–H stretching of quinolinone at 3188 and 3424 cm^−1^, aromatic C–H stretching peak at 3035 cm^−1^, aliphatic C–H stretching peaks at 2788 to 3000 cm^−1^, stretching of aryl ketone C=O at 1668 cm^−1^, N-H tetrazole bending at 1505 cm^−1^, and aryl alkyl ether C–O stretching vibration at 1243 cm^−1^ (asymmetric) and 1038 cm^−1^ (symmetric) [73,74].

FTIR analysis confirmed the compatibility between CIL and the formulation components, as the positions of characteristic peaks of CIL (1668, 1505 and 1243 cm^−1^) were not altered. These findings suggested no major chemical interaction between CIL and formulation excipients, indicating the stability of CIL within the spanlastic carrier. Moreover, the presence of these characteristic peaks supports CIL-SPA formulation prepared by using 400 mg Span^®^ 60, 100 mg Tween^®^ 80, and 1% *w*/*v* oleic acid, which formed a multi-layered membrane and encapsulated CIL within the lipophilic layer and the lipophilic penetration enhancer (oleic acid), resulting in reduced drug leakage and increased EE%.

### 2.4. Dynamic Light Scattering Measurement Results

Average hydrodynamic diameter (Z-average), polydispersity index (PDI), and zeta potential (ZP) are significant parameters influencing the brain targeting, bioavailability, and stability of spanlastics [75]. As reported in the literature, the ideal Z-average for brain targeting (through olfactory and trigeminal nerve pathways) and for increasing the blood circulation time is in the range of 100 to 200 nm [76,77]. However, nanoparticles with a Z-average < 300 nm were able to cross the olfactory pathway and to be taken up into the brain [78,79,80]. PDI value can be used for describing the degree of uniformity in size distribution of nanoparticles. It ranges from 0 (monodisperse vesicles) to 1 (polydisperse vesicles), where PDI values < 0.3 indicate homogenous vesicles [81]. However, PDI values < 0.5 reported to be acceptable for homogenous size distribution [81,82]. ZP refers to the electrokinetic potential in colloidal systems. It is an important parameter to evaluate the pharmacokinetics, phagocytosis in the bloodstream, and physical stability of the nanoparticles [83]. Nanoparticles with a ZP value > |±30| mV are considered physically stable due to the electrostatic repulsive forces that prevent particle aggregation [84].

As shown in Table 1, Z-average increased by increasing the amount of Tween^®^ 80 as a nonionic edge activator, which is suitable for decreasing interfacial tension and increasing flexibility of the bilayer membranes, thus increasing the elasticity and reducing the deformability of the spanlastic vesicles, and as a result, increasing the water uptake, which leads to an increased Z-average [36]. Similar results were reported by previous studies [44,85]. PDI values ranged from 0.20 ± 0.02 to 0.37 ± 0.04, indicating a relatively homogeneous distribution. CIL-SPAs showed a negative ZP ranging from −27.80 ± 0.35 to −61.67 ± 0.21 mV. The highest ZP values mean the highest stability. Based on the results, CIL-SPA1 was selected as the optimal formulation because it had the lowest Z-average, with a narrow size distribution and the highest negative ZP value.

Furthermore, the prepared in situ gels showed a Z-average < 300 nm with acceptable PDI values < 0.5. The negative ZP of SPA-PG and SPA-P407 could help in the interaction with mucin in nasal mucosa (mucin binding, hydrogen bonds), while SPA-CS showed a positive ZP value due to the presence of chitosan, which interacts with anionic sialic and acidic moieties of glycosaminoglycan in mucin [86]. Similar results were obtained by Zaki et al. (2022) who prepared brigatinib-loaded spanlastics using Span^®^ 60 and Tween^®^ 80 in a ratio of 4:1, which showed the lowest particle size. The optimized formulation had a Z-average value of 388 nm, PDI value of 0.474, and ZP value of −29.6 mV, while Z-average increased to 395.4 nm after coating with chitosan [44]. Sayyed et al. (2024) developed lacosamide-loaded spanlastics for a nose-to-brain delivery system using Span^®^ 60 and Tween^®^ 80 (80:20% *w*/*w*) and different types of penetration enhancers (PEs). Their study showed that Z-average was smaller in case of lipophilic PEs than the water-soluble PEs [41]. The results indicate the suitability of CIL-SPA and CIL-SPA-loaded in situ gelling formulations for nose-to-brain delivery.

### 2.5. Droplet Size Distribution

Droplet size distribution is an important indicator of in vitro bioavailability and bioequivalence for the liquid nasal formulation, and it is recommended by US FDA and EMA to evaluate the suitability for spraying into the nasal cavity [87,88]. The typical droplet size ranges between 20 and around 200 µm [89]. As reported in the literature, droplets with a median volume distribution (Dv50) of <10 µm are strongly influenced by the airflow and could be inhaled through the nasopharynx and reach the lungs [90], or they could be deposited in the anterior region of the nasal cavity (the vestibular area), which enhances the formulations’ clearance from the nasal cavity [87]. In this study, we used the manual actuation of each nasal spray to simulate the real clinical situation. The results of the tested formulations (CIL-SPA, SPA-PG, SPA-P407, SPA-CS) indicated that all formulations were suitable for nasal administration (Dv50 values ranged from 70.16 ± 2.70 to 98.58 ± 3.43 µm) and can avoid the deposition in the lower respiratory system, which increases the absorption through the nasal cavity (olfactory region; the target site for direct access to CNS [91,92]). Droplet size spread (Span) values are used to evaluate the width of droplet size distribution. All prepared formulations had low span values with no significant effect of the gel base. As presented in Table 2, there was no significant effect (*p* > 0.05) on 10% of the cumulative volume distribution (Dv10), Dv50, 90% of the cumulative volume distribution Dv90, and span values of CIL-SPA after loaded into in situ gelling matrices of Phytagel^®^ (SPA-PG) and Poloxamer-407 (SPA-P407), while in the case of chitosan (SPA-CS), there was a significant increase in Dv50 and Dv90 values (** *p* < 0.01 and *** *p* < 0.001, respectively). This result can be explained by the fact that Phytagel^®^ and Poloxamer-407 are liquid at room temperature and convert into a viscous gel in the nasal cavity.

### 2.6. Encapsulation Efficiency (EE%) and Loading Capacity (LC%)

**Figure 1:**
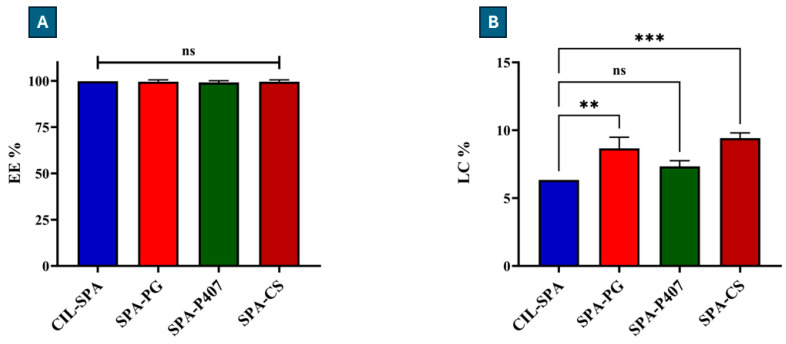
(**A**): EE% and (**B**): LC% results of the prepared formulations; (ns) means marks non-significant, whereas asterisks indicate significant change (** *p* < 0.01 and *** *p* < 0.001). Results are expressed as means ± SD (n = 3).

CIL was successfully encapsulated into spanlastic vesicles. According to the literature, the nano-carrier system is considered efficient for drug delivery if the EE% is >50% and LC% is >5% [93]. CIL-SPA (Figure 1) showed a high EE % (ca. 100%) and good LC% (6.34%). The composition of spanlastic vesicles has a significant impact on the EE%. Span^®^ 60 has a HLB value of about 4.7, long chain length (C18), and high transition temperature (53–55 °C), which result in reduced drug leakage from the lipophilic layer and in an increased EE% [94]. In addition, elevating the amount of Span^®^ 60 improves the formation of a multi-layered membrane, thus increasing EE% [95]. Moreover, CIL has a high solubility in oleic acid [96], which could help to encapsulate CIL within the lipophilic layer and prevent the leakage to the external aqueous environment. Tween^®^ 80 was used in a low amount, and, as reported in previous studies [85], reducing the amount of Tween^®^ 80 leads to an increased EE%. In addition, Tween^®^ 80 has an HLB value of 14.9, reflecting higher hydrophobicity and improved membrane integrity, resulting in a higher EE% [97]. All prepared formulations had a high EE% (ca. 100%) and acceptable LC% values (>5%). SPA-PG and SPA-CS showed a significant increase in LC% compared to CIL-SPA (** *p* < 0.01 and *** *p* < 0.001, respectively). These results indicate the efficiency of the vesicles as drug carriers for CIL.

### 2.7. Rheological Evaluation

Rheological properties (viscosity and elasticity) of the nasal formulations have an important impact on the efficacy due to extending the nasal residence time. SPA-PG samples showed strong gelation after mixing the samples with SNES, which resulted in the formation of a dense gel structure and a separated liquid. The measurements were made on the concentrated gel, leading to very high viscosity values.

**Figure 2:**
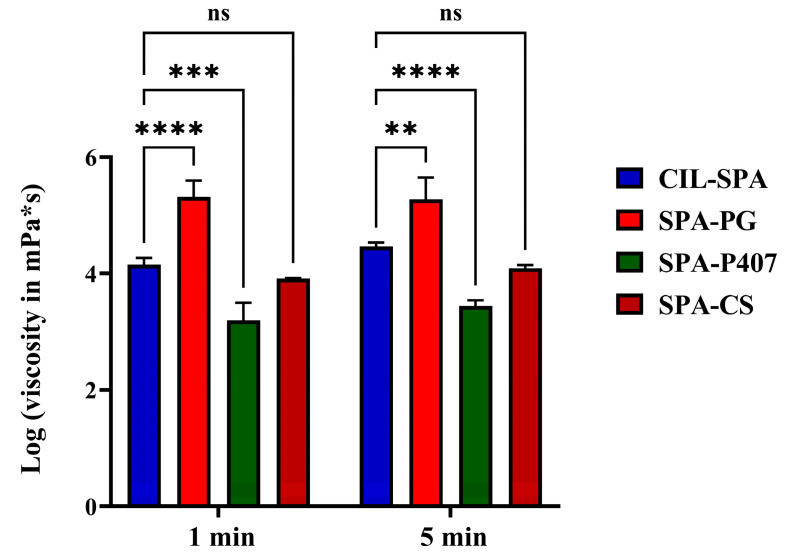
log (viscosity in mPa*s) results of the prepared formulations. Results are expressed as means ± SD (n = 3) (ns: non-significant change, ** *p* < 0.01, *** *p* < 0.001 and **** *p* <0.0001).

The viscosity evaluation of all formulations showed that the SPA-PG sample had a significantly higher viscosity value after 1 and 5 min compared to CIL-SPA (**** *p* < 0.0001 and ** *p* < 0.01, respectively) and a higher viscosity value than other formulations. The time did not show a significant effect (*p* > 0.05) on viscosity values of all prepared formulations which, after 1 and 5 min, were the same (Figure 2).

The prepared formulations showed higher storage modulus (G′) values in comparison to loss modulus (G″) values, indicating that polymer chains are capable of forming a gel network, and they have mainly elastic behavior [98] (Table 3). SPA-PG had significantly higher G′ and G″ values (**** *p* < 0.0001) compared to other formulations, indicating that SPA-PG had the strongest elastic properties.

In addition, the loss factor (tan δ) is another parameter to evaluate the elasticity of the formulations. A lower value of tan δ indicates higher elasticity. A value < 1 indicates a solid-like behavior (elastic), while value > 1 indicates a liquid-like gel behavior [99]. For SPA-PG, the formed solid gel structure resulted in higher elasticity compared to the other formulations, which was also confirmed by the low tan δ value (0.17 ± 0.09). CIL-SPA, SPA-P407, and SPA-CS showed a very similar rheological character when mixed with SNES. A weak gel structure characterizes all of them, which is also well demonstrated by low G’ and loss tangent values between 0.2 and 1. Summarizing the rheological characteristics, all samples showed gel characteristics and the loss tangent was less than 1, but the SPA-PG sample reacted with significant gelation to the addition of nasal fluid; this sample showed the strongest gel characteristics.

### 2.8. Mucoadhesion Results

**Figure 3:**
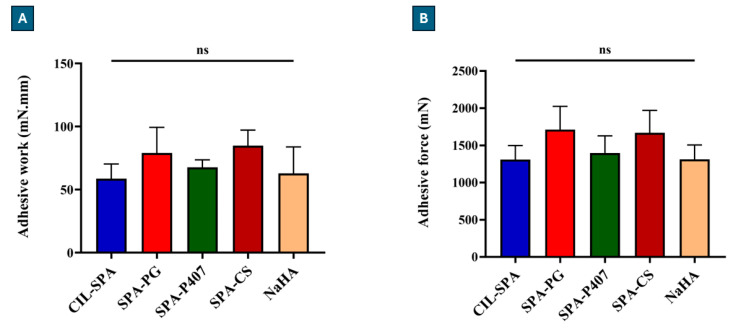
The adhesion work (**A**) and adhesive force (**B**) of the prepared formulations in comparison to NaHA (0.5% *w*/*v*). Results are expressed as means ± SD (n = 5).

The adhesive force and adhesive work of the prepared formulations were evaluated in comparison to 0.5% *w*/*v* sodium hyaluronate solution (NaHA) with SNES to mimic nasal conditions to evaluate the mucoadhesiveness, thus overcoming the nasal clearance (Figure 3).

Analysis of variance (ANOVA) showed that the difference in adhesive force and adhesive work was not statistically significant compared to NaHA (0.5% *w*/*v*), which is a mucoadhesive polymer commonly employed for nasal formulations [100]. Hence, the prepared formulations exhibited good mucoadhesion properties, which are essential to prolong nasal residence time and enhance the absorption.

In terms of mucoadhesion, due to the rheological characteristics, the most remarkable mucoadhesion was expected in the case of the SPA-PG samples, but despite the weak gel structure, SPA-CS samples also showed similar values (higher adhesive force and work values). The mucoadhesiveness of chitosan is well known, which is explained in the literature by electrostatic attraction and hydrogen bonding, among others. Accordingly, in the case of chitosan, even with a weaker gel structure, better mucoadhesivity can be expected, and it was verified in our measurements as well.

### 2.9. In Vitro Drug Release Studies

**Figure 4:**
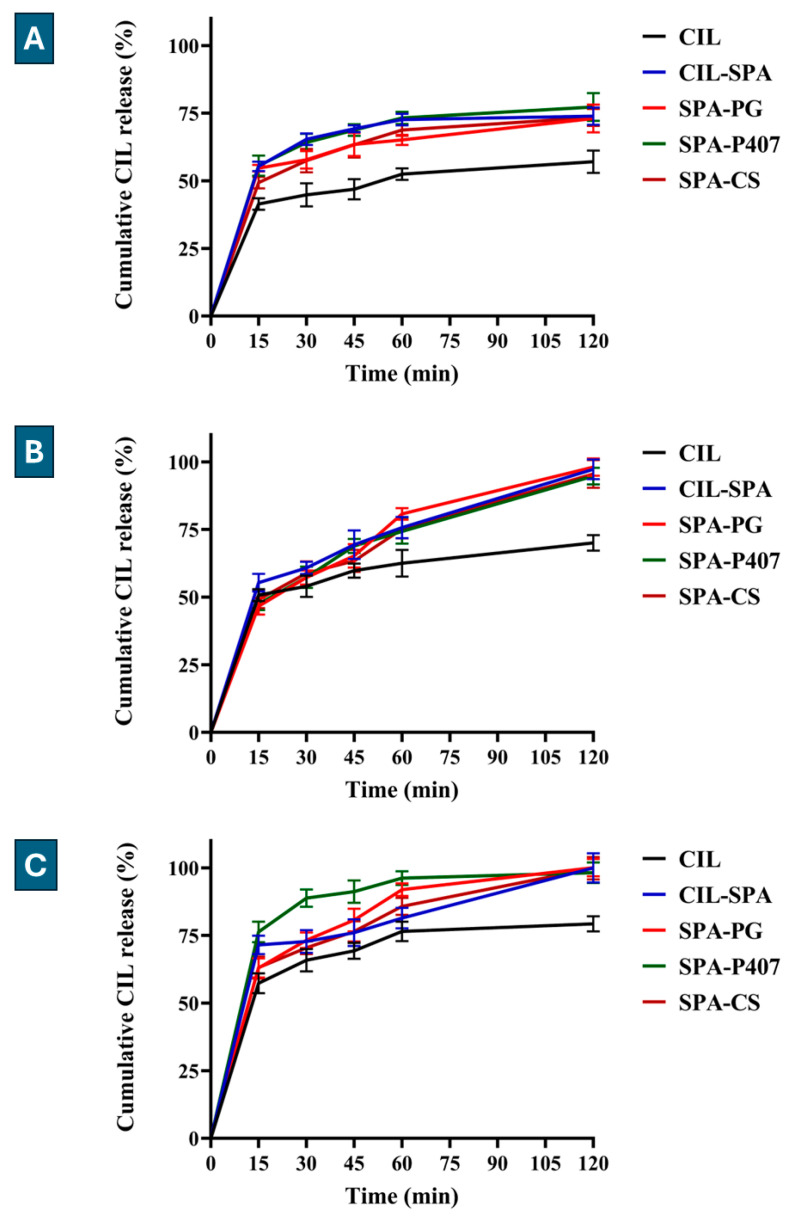
Release profile of the prepared formulations compared to pure CIL. (**A**): in SNES (pH 5.6), (**B**): in PBS (pH 7.4), and (**C**): in SCSF (pH 7.4). Results are presented as means ± SD (n = 3).

Drug release is an important biopharmaceutical parameter for evaluating the efficiency of the drug delivery system compared to the initial drug. CIL is a BCS class II drug; therefore, the release is the rate-limiting step for the absorption from the site of the administration. As shown in Figure 4, the release results in SCSF (pH 7.4) showed a higher release rate compared to PBS (pH 7.4) and SNES (pH 5.6), which could be explained by the influence of release medium composition on the release rate, where the increase in sodium content leads to an increase in the release rate (SCSF > PBS > SNES), and the cations in SCSF may influence the drug diffusion through the gel matrix, as reported by Mohanraj et al. (2013) [101].

The results showed that the prepared formulations released ca. 75% of CIL in SNES after 2 h, while pure CIL released ca. 57% (Figure 4A). After 15 min, SPA-CS showed a significant increase in the release rate (*p* < 0.05), while CIL-SPA, SPA-PG, and SPA-P407 showed higher significant release rates (*p* < 0.0001). Then, after each time point, the formulations showed higher significant release rates (*p* < 0.0001) in comparison to pure CIL.

The formulations released ca. 100% of CIL in PBS after 2 h, while pure CIL released only 70.05 ± 2.40% (Figure 4B). After 30 min, only CIL-SPA showed a significant increase in the release rate (*p* < 0.05); then, after 45 min, CIL-SPA and SPA-P407 showed a significantly higher dissolution (*p* < 0.001), while after 60 and 120 min, all formulations exhibited a substantially higher release rate (*p* < 0.0001).

In addition, the prepared formulations released ca. 100% of CIL in SCSF after 2 h, while pure CIL released ca. 80% (*p* < 0.0001) (Figure 4C). After 15 min, only CIL-SPA and SPA-P407 showed higher significant release rates (*p* < 0.0001), then after 30 min, only SPA-P407 showed higher significant release rates (*p* < 0.0001). After 45 min, SPA-PG and SPA-P407 showed higher significant release rates (*p* < 0.01 and *p* < 0.0001, respectively). Then, after 60 min, SPA-PG, SPA-P407, and SPA-CS showed higher significant release rates (*p* < 0.0001, *p* < 0.0001, and *p* < 0.05, respectively) in comparison to pure CIL.

We can explain this release rate difference by the effect of Span^®^ 60, Tween^®^ 80, and the nanosized vesicles, which increased the surface area; as a result, the hydrophilic properties improved, and the release rate of CIL increased. Furthermore, the release rate from the in situ gelling formulation depends on the type of polymer.

The type of polymer influences the drug release rate by the diffusion from the gel matrix, which depends on the shape, structure, or integrity of the matrix, or by gel erosion, which leads to large pores in the gel matrix, and thus, water uptake into the polymer [102]. The results demonstrated that the release rate had a diffusion-erosion type, where we can notice a biphasic release behavior. The first phase is basically due to the drug diffusion through the gel matrix (fast release within 15 min), while the second phase is the release of the remaining amount of CIL due to gel erosion (bulk erosion and surface erosion [103]). The in situ gel formulations (SPA-PG, SPA-P407, SPA-CS) showed a non-significant difference in the release profile, which could be due to the effect of Span^®^ 60 and Tween^®^ 80.

Zaki et al. (2022) showed that the chitosan-coated spanlastic formulation (Z-average was 395.4 nm) had a higher release rate than the pure drug in PBS (pH 7.4), with a biphasic release behavior [44].

The DDsolver^®^ software was used to evaluate the release kinetics for the prepared formulations. The model with the highest R^2^ value, low Akaike Information Criteria (AIC), and high Model Selection Criterion (MSC) values is the best kinetics model [104,105,106]. Tables S1–S3 illustrated that the release kinetics profiles for CIL-SPA, SPA-PG, SPA-P407, and SPA-CS were fitted by the Korsmeyer–Peppas kinetic model in SNES, PBS, and SCSF, with release exponent “n” values < 0.45, indicating a quasi-Fickian diffusion [107].

### 2.10. In Vitro Permeability Measurements

#### 2.10.1. Horizontal Diffusion Cell Measurement

**Figure 5:**
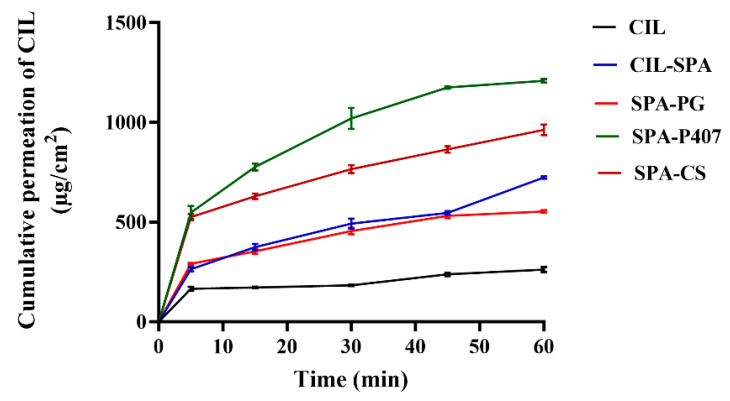
The cumulative permeation of CIL for the prepared formulations in comparison to pure CIL. Results are presented as means ± SD (n = 3).

The modified Side-Bi-Side^®^-type horizontal diffusion cell is a useful in vitro method to evaluate the permeation of CIL at simulated nasal conditions (pH 5.6). Figure 5 illustrates the cumulative amount of CIL permeated (P_app_) through the impregnated artificial membrane (mimicking nasal conditions) compared to initial CIL in the cases of various prepared formulations. The results demonstrated that the permeation of CIL through the impregnated artificial membrane was significantly higher in the cases of CIL-SPA, SPA-PG, SPA-P407, and SPA-CS compared to pure CIL at each time point (*p* < 0.0001). Among the formulations, SPA-PG showed the lowest permeation rate as an effect of SNES on the formation of a dense gel structure, thereby hindering drug diffusion.

The penetration efficacy of CIL was 8.72%, while for the prepared samples, the values were 24.12%, 18.43%, 40.26%, and 32.07% for CIL-SPA, SPA-PG, SPA-P407, and SPA-CS, respectively.

**Figure 6:**
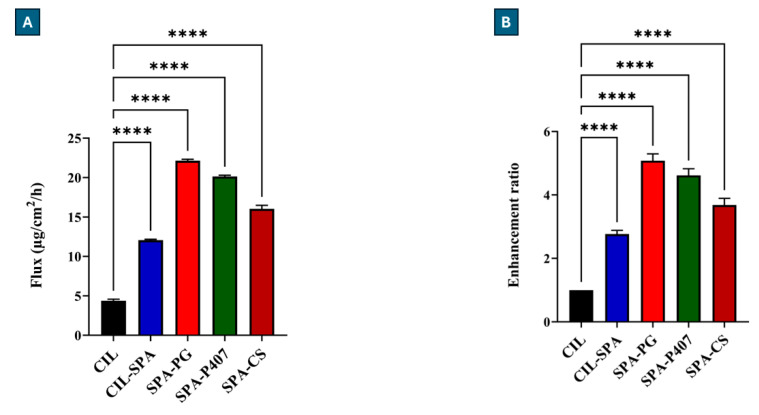
The steady-state flux (**A**) and enhancement ratio (**B**) of the prepared formulations in comparison to pure CIL at SNES condition. Results are expressed as means ± SD (n = 3) (**** *p* <0.0001).

In addition, the evaluation of the biopharmaceutical parameters (Figure 6) showed that the value of steady-state flux (J_ss_) and the enhancement ratio (ER) of prepared formulations were significantly higher than in case of initial CIL (*p* < 0.0001). This result indicates the improvement in CIL solubility and permeability by using spanlastic vesicles and spanlastic-loaded in situ gelling formulations.

#### 2.10.2. Blood–Brain Barrier Parallel Artificial Membrane Permeability Assay (BBB-PAMPA)

**Figure 7:**
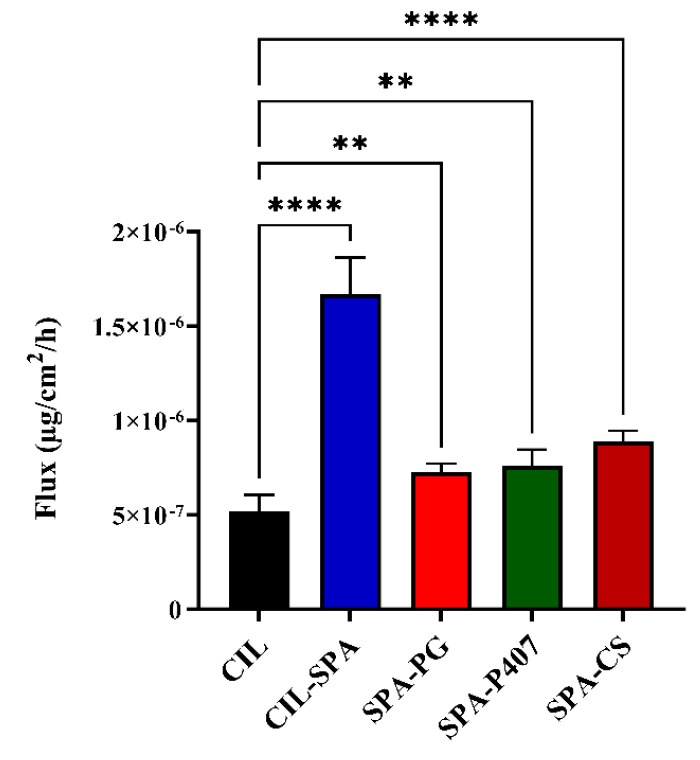
Flux values of the prepared formulations in comparison to pure CIL. Results are expressed as means ± SD (n = 6) (** *p* < 0.01 and **** *p* <0.0001).

BBB-PAMPA is a useful in vitro test to evaluate the CIL permeability (passive transport) through the porcine brain polar lipid extract [108,109], which is one of the major absorption routes of lipophilic molecules through the nasal mucosa and the BBB [110,111]. The results demonstrated that all prepared formulations had significantly higher flux values than pure CIL (as shown in Figure 7). This result can be explained by the effect of the formulations’ composition (Span^®^ 60, Tween^®^ 80, and oleic acid), which improved the flux through the brain lipid by increasing the solubility of CIL. SPA-CS had higher flux value compared to SPA-PG and SPA-P407, which could be related to its positive charge.

### 2.11. Biological Stability Test Evaluation

**Figure 8:**
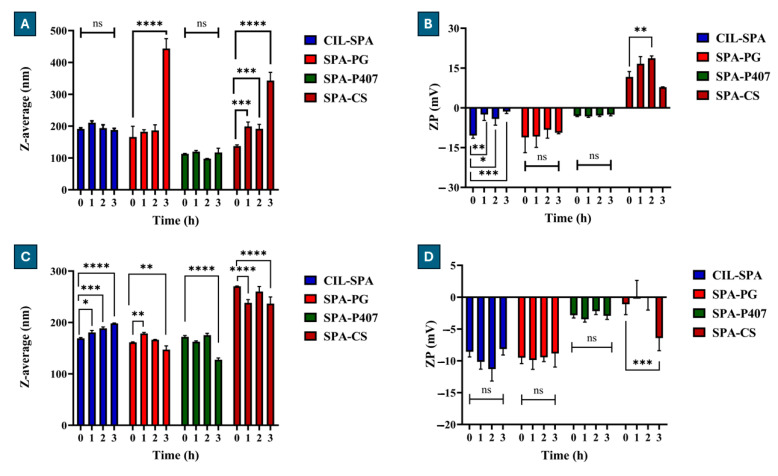
Biological stability results for the prepared formulations, (**A**): Z-average in SNES, (**B**): ZP in SNES, (**C**): Z-average in SCSF, (**D**): ZP in SCSF. Results are expressed as means ± SD (n = 3) (ns: non-significant change, * *p* < 0.05, ** *p* < 0.01, *** *p* < 0.001 and **** *p* <0.0001).

The biological stability test in SNES and SCSF is a useful parameter to evaluate the stability of the vesicles after the intranasal administration. In this study, we used the colloidal parameters as Z-average and ZP as physical indicators for the stability. As shown in Figure 8A, CIL-SPA and SPA-P407 showed no significant changes in Z-average after 3 h of incubation with SNES (and remained within the acceptance range < 300 nm), while SPA-PG showed a high significant increase in Z-average after 3 h, which could be due to the formation of gel (>300 nm), and SPA-CS showed a significant increase in Z-average after each time point (>300 nm). Moreover, Figure 8B demonstrated that only SPA-PG and SPA-P407 had no significant changes in ZP, while CIL-SPA showed significant changes during the test.

The stability results in SCSF showed that all the prepared formulations remained within the acceptance range of Z-average < 300 nm, which could suggest that the vesicles were more stable in SCSF than SNES. CIL-SPA showed a significant increase in Z-average after each time point (Figure 8C), while SPA-PG, SPA-P407, and SPA-CS showed a significant decrease in Z-average after 3 h. Furthermore, Figure 8D demonstrated that CIL-SPA, SPA-PG, and SPA-P407 had no significant changes in ZP in the end of the test, while SPA-CS showed a significant increase in the absolute value of ZP.

The results indicate that the composition and pH of the medium had a significant impact on the vesicles.

## 3. Conclusions

The nose-to-brain delivery of CIL with a suitable drug delivery system is a promising approach. To ensure the therapeutic effect, the drug carrier should present several advantages, including EE% > 50% and LC% > 5%, homogeneous nanosized particles (PDI < 0.5 and Z-average < 300 nm to cross the olfactory region and reach the brain and improve the solubility), ZP > |±30 mV| (to ensure the physical stability, bypass BBB, and escape from the reticuloendothelial system), good viscosity and mucoadhesion properties (to prolong the residence time in the nasal cavity, which improves the absorption and permeability through the nasal mucosa), and acceptable droplet size distribution (Dv50 ranges between 20 and around 200 µm to ensure high deposition in the olfactory region).

The present study revealed that CIL was suitable for successful encapsulation in spanlastic vesicles intended for nose-to-brain delivery. The optimized composition consisted of 400 mg of Span^®^ 60, 100 mg Tween^®^ 80, and 1% *w*/*v* oleic acid as a penetration enhancer, which was incorporated into various in situ gelling matrices to extend residence time in the nasal cavity and to improve the absorption into the brain. The developed in situ gels (SPA-PG, SPA, P407, SPA-CS) showed nanosized vesicles (<300 nm), acceptable PDI values (<0.5), and ZP (>|±30| mV) with high EE% (>99%) and LC% (>5%), and these properties make them suitable and effective drug carriers for nose-to-brain delivery. All formulations exhibited adequate mucoadhesion properties, which make them resist nasal clearance and improve the nasal absorption. Biopharmaceutical evaluations revealed that in situ gelling formulations improved the drug release and permeability behavior both at nasal and BBB conditions. Therefore, according to our results, we can conclude that both CIL-SPA and the developed in situ gelling formulations could be promising drug carriers for nose-to-brain delivery of CIL, which may contribute to a significantly improved bioavailability of the drug.

## 4. Materials and Methods

### 4.1. Chemicals

CIL was provided by Egis Pharmaceuticals Plc. (Budapest, Hungary). Methanol 99.99% *v*/*v* (HPLC grade), ortho-phosphoric acid 85% *v*/*v* (HPLC grade), dimethyl sulfoxide (DMSO), and mannitol were purchased from Molar Chemicals Ltd. (Budapest, Hungary). Acetonitrile 99.8% *v*/*v* (HPLC grade) was purchased from LGC PromoChem GmbH (Budapest, Hungary). Dodecane, hexane, Span^®^ 60, Tween^®^ 80, Phytagel^®^, chitosan (low molecular weight 50,000–190,000 Da), Poloxamer-407, EtOH 96% *v*/*v*, and oleic acid were purchased from Merck KGaA (Darmstadt, Germany). Polar brain lipid extract and porcine stomach mucin were acquired from Sigma Aldrich Co., Ltd. (Budapest, Hungary). The phosphate buffer solution (PBS pH 7.4) was freshly prepared, which consists of 8.00 g/L sodium chloride (NaCl), 0.20 g/L potassium chloride (KCl), 1.44 g/L Na_2_HPO_4_·2H_2_O, and 0.12 g/L KH_2_PO_4_ dissolved in 1000 mL of deionized water at pH 7.4 [112]. SNES was freshly prepared, consisting of 8.77 g NaCl, 2.98 g KCl, and 0.59 g anhydrous calcium chloride (CaCl_2_) dissolved in 1000 mL of deionized water at pH 5.6 [113]. The simulated cerebrospinal fluid (SCSF) was freshly prepared, which consists of 0.2 M NaCl, 0.02 M NaHCO_3_, 2 mM KCl, 0.5 mM KH_2_PO_4_, 1.2 mM CaCl_2_, 1.8 mM MgCl_2_, 0.5 mM Na_2_SO_4_, and 5.8 mM D-glucose at pH 7.4 [101]. These chemicals were obtained from Sigma-Aldrich Co., Ltd. (Budapest, Hungary). In all experiments, the purified water was produced using Milli-Q^®^ Gradient Water Purification System (Merck Ltd., Budapest, Hungary).

### 4.2. Preparation of Cilostazol-Loaded Spanlastic Formulations (CIL-SPAs)

CIL-SPAs were prepared using a modified EtOH injection method, using Span^®^ 60 as vesicle builder, Tween^®^ 80 as edge activator, and oleic acid 1% *w/v* as penetration enhancer [43]. Preliminary studies were conducted to select the optimal amount of Span^®^ 60 and Tween^®^ 80 (as shown in Table 4). Briefly, CIL, Span^®^ 60, and oleic acid 1% *w*/*v* were dissolved in 2 mL of EtOH using an ultrasonication bath (40 kHz for 10 min at 60 °C) and injected dropwise into 10 mL of a preheated aqueous solution (distilled water, 60 °C) containing Tween^®^ 80. Then, it was stirred for 30 min at 800 rpm and followed by sonication (at 80% amplitude for 2 min and 0.5 cycles) to prepare the spanlastic vesicles. Subsequently, the final volume was adjusted to 10 mL with purified water. Finally, the resulting formulations were kept overnight in the refrigerator (5 ± 0.5 °C) to allow the formation of the bilayer. Aluminum foil was used during preparation, storage, and further investigations.

### 4.3. Preparation of Cilostazol Spanlastic-Loaded In Situ Gel

The optimal CIL-SPA formulation was selected according to the Z-average less than 300 nm, the PDI less than 0.5, and the ZP higher than |±30| mV, and then transformed to a nanogel using the following gel bases: Phytagel^®^, Poloxamer-407, and chitosan. Firstly, Phytagel^®^ was dissolved in preheated distilled water (90 °C) under constant stirring (800 rpm) until a homogeneous solution formed, then cooled to room temperature, followed by adding the optimal CIL-SPA in a 1:1 *v*/*v* ratio, and mixed at room temperature until homogeneity to prepare the SPA-PG formulation (the final concentration of Phytagel^®^ was 0.2% *w*/*v*) [114,115]. Secondly, Poloxamer-407 was dissolved in distilled water at room temperature under constant stirring (800 rpm) until a homogeneous solution formed and then stored in the refrigerator at 4 °C overnight [116], followed by adding the optimal CIL-SPA in a 1:1 *v*/*v* ratio, and mixed at room temperature until homogeneity to prepare the formulation SPA-P407 (the final concentration of Poloxamer-407 was 12% *w*/*v*). Finally, chitosan was dissolved in a 1% *v*/*v* aqueous acetic acid solution overnight at room temperature with constant stirring (800 rpm) until a homogeneous solution formed, then the pH was adjusted to 4.5 with NaOH 0.1 M and filtered using a 0.45 µm syringe filters [117], followed by adding the optimal CIL-SPA in a ratio of 1:1 *v*/*v*, and mixed at room temperature until homogeneity to prepare the SPA-CS formulation (the final concentration of chitosan was 0.5% *w*/*v*).

### 4.4. Lyophilization

Freeze-dried formulations were prepared in a Scanvac, CoolSafe 100-9 Pro-type apparatus (LaboGeneApS, Lynge, Denmark). A total of 1.5 mL of the formulations was lyophilized by adding 5% *w*/*v* mannitol as a cryoprotectant. Freeze-drying took place at −40 °C for 12 h at 0.013 mbar pressure with an additional secondary drying of 3 h at 25 °C. The process was controlled by Scanlaf CTS16a02 software. The samples were then stored in the refrigerator until further investigation. Freeze-dried samples were used for FTIR measurements and the BBB-PAMPA test.

### 4.5. FTIR Measurements

FTIR spectra of initial CIL, excipients, blank formulations (without CIL), and the prepared formulation (CIL-SPA, SPA-PG, SPA-P407, and SPA-CS) was performed using AVATAR 330 FT-IR instrument (Thermo Nicolet, Unicam Hungary Ltd., Budapest, Hungary) equipped with a deuterated triglycine sulfate detector. Each sample was scanned 128 times the spectral range of 400–4000 cm^−1^ with a spectral resolution of 4 cm^−1^. Spectral evaluations were performed using OriginPro 8.6 software (OriginLab Corporation, Northampton, MA, USA).

### 4.6. Z-Average, PDI, and ZP Determination

The Z-average, PDI, and ZP values of the formulations were measured by dynamic light scattering (Malvern Instrument Ltd., Worcestershire, UK). The measurements were carried out using disposable folded capillary cells at room temperature and a scattering angle of 173° using a He-Ne laser of 633 nm with a refractive index of 1.76. The formulations were diluted in distilled water (1:10 *v*/*v*) using an ultrasonic bath for 4 min. All measurements were carried out in triplicate. The results were presented as means ± SD.

### 4.7. Droplet Size Distribution Measurment

The droplet size distribution of the formulations was determined with the laser diffraction method (Malvern Spraytec^®^, Malvern Instruments Ltd., Malvern, UK), with a 300 mm lens for analyzing droplet size in the range of 0.1–900 μm (Dv50: 0.5–600 μm). The tip of the nasal spray device was positioned horizontally to the receiving lens to reach the laser beam precisely in the center of the expansion of the spray cone. Prior the measurement, the formulations were transferred into a nasal spray container. Three parallel measurements were performed with each nasal spray formulation at room temperature. Measurement data were analyzed with the Spraytec^®^ software v4.00 (Malvern Panalytical Ltd., Malvern, UK), and the volume diameter of 10% (Dv10), 50% (Dv50), and 90% (Dv90) of the cumulative volume distribution was determined. The results were presented as means ± SD. The Span was determined using the following equation [118]:

(1) $$Span=\frac{\left(Dv90-Dv10\right)}{Dv50}$$

### 4.8. Drug Content, Encapsulation Efficiency (EE%), and Loading Capacity (LC%)

The drug content was determined by dissolving 1 mL of the prepared formulation in 4 mL of methanol using an ultrasonication bath for 10 min, filtered using 0.45 µm syringe filters, and CIL concentration was analyzed with HPLC. The stationary phase was Zorbax^®^ SB-CN C18 column (5 μm, 150 mm × 4.6 mm, 100 Å) and the mobile phase consisted of acetonitrile:methanol:water in the ratio 50:20:30, *v*/*v*. The 10 µL samples were injected and elution was performed with a 0.5 mL/min flow rate. Chromatograms were detected at 260 nm using a UV-VIS diode array detector.

EE% was determined using a Hermle Z323 laboratory centrifuge (Hermle AG, Gossheim, Germany). A total of 1 mL of the prepared samples was filled in an Eppendorf tube and centrifuged at 15,000 rpm for 30 min at 4 ± 1 °C [119]. Then, the clear supernatant solutions were filtered and analyzed using the same HPLC system. EE% and LC% were determined using the following equations [119,120]:

(2) $$EE\%=\frac{C_{Total}-C_{free}}{C_{Total}}\times 100$$

(3) $$LC\%=\frac{Mass\;of\;drug\;encapsulated}{Mass\;of\;formulation\;components}\times 100\%$$

where C_total_ is the initial concentration of CIL in the formulation, and C_free_ is the concentration of CIL in the supernatant after centrifugation. All measurements were performed in triplicate, and the results were presented as means ± SD.

### 4.9. Rheological Studies

The rheological properties were measured with a Physica MCR302 rheometer (Anton Paar, Graz, Austria). A cone-and-plate-type measuring device was used with a gap height in the middle of the cone of 0.046 mm, a diameter of 25 mm, and a cone angle of 1°. The samples were prepared by gentle manual mixing of the formulations with SNES in a 1:1 ratio. The mixture was measured using a steady-shear viscosity measurement at a constant shear rate of 1 Hz, which corresponds to the shear rate of nasal mucus [121]. The viscosities of the samples were compared after 1 and 5 min. After the steady-shear viscosity measurement, the viscoelastic behavior of SNES-samples was analyzed by means of oscillation measurements. The frequency sweep measurement was performed, and the G′ and G″ were measured at a constant strain of 0.2%, which was in the linear viscoelastic region of the samples. The values of G′ and tan δ values at 10 Hz were used for further comparison (The directional movement of the nasal epithelium cilia has a beat frequency of 10 Hz) [121]. All measurements were carried out at 35 °C in triplicate, and the results were presented as means ± SD. The tan δ was calculated using the following equation:

(4) $$\tan\delta =\frac{G^{\prime \prime }}{G^{\prime }}$$

### 4.10. Tensile Tests

For tensile tests, a TA-XT Plus Texture analyzer (Metron Kft, Budapest, Hungary) instrument connected with a 5 kg load cell and a 1 cm diameter cylinder was applied. A total of 20 mg of the samples were placed on a filter paper fixed to the cylinder probe. The probe was then pressed to a filter paper pretreated with 50 µL of 8% *w*/*w* mucin dispersion as a simulated mucosal membrane. The mucin dispersion was prepared in SNES. After setting the preload of 2500 mN for 3 min, the cylinder probe was moved upwards to detach the samples from the artificial mucosa. The maximum adhesive force (detachment force) was registered and the adhesive work (A, mN·mm) calculated based on the area (AUC) under the “force versus distance” curve. As a reference, aqueous solution of sodium hyaluronate (NaHa) (0.5% *w*/*v*) was used. Five parallel measurements were performed at 35 °C [113]. The results were presented as means ± SD.

### 4.11. In Vitro Drug Release Tests

The CIL release from the prepared formulations was evaluated using the dialysis method. Formulations with a certain number of formulations (equivalent to 3 mg/mL) were pipetted into presoaked dialysis bags (Spectra/Por^®^, MWCO 12–14 kDa, Spectrum Laboratories Inc., Rancho Dominguez, CA, USA) and then transferred to the Hanson SR8 Plus paddle apparatus (Teledyne Hanson Research, Chatsworth, CA, USA). The test was performed in SNES (pH 5.6), PBS (pH 7.4), and SCSF (pH 7.4) The volume of the release medium was 100 mL, and the stirring speed was 50 rpm at 35 ± 0.5 °C. Aliquots were withdrawn at defined time points up to 2 h and then injected into the HPLC to determine the concentration of CIL. Measurements were performed in triplicate. The results were presented as means ± SD.

The model-dependent approaches (including zero order, first order, Higuchi model, and Korsmeyer–Peppas model) were used to evaluate the release kinetics of CIL samples. The DDsolver^®^ add-in software (a menu-driven add-in program for Microsoft Excel) was used for the mathematical evaluation of the release kinetics, and the fit of each model was determined by comparing the rate constant (K), correlation coefficient (R^2^), Akaike Information Criterion (AIC), and Model Selection Criterion (MSC) [104,105,122,123].

### 4.12. In Vitro Permeability Studies

#### 4.12.1. Side-Bi-Side-Type Horizontal Diffusion Cell System

The cumulative permeability of CIL through the artificial nasal mucosa was evaluated using a modified Side-Bi-Side^®^-type horizontal diffusion system [124,125]. A synthetic membrane (PALL Metricel membrane with 0.45 µm pores) was impregnated with isopropyl myristate for 30 min prior to investigation and placed between the donor and acceptor phases to provide a suitable surface for the permeability study. The donor chamber consisted of 8 mL of SNES and 1 mL of each formulation and pure CIL (with a nominal concentration of 3 mg/mL), while the acceptor chamber contained 9 mL of PBS solution (pH 7.4). The temperature of both chambers was maintained at 32 ± 0.5 °C using a thermostat (ThermoHaake C 10-P5, Sigma–Aldrich Co. Ltd., Budapest, Hungary). A continuous stirring at 300 rpm was applied both in the donor and acceptor compartments. Aliquots from the acceptor phase (150 µL) were withdrawn at 5, 15, 30, 45, and 60 min and replaced with fresh PBS. Then, CIL concentration was determined using the same HPLC system. The apparent permeability (P_app_), steady-state flux (J_ss_), and the enhancement ratio (ER) were calculated using the following equations:

(5) $$P_{app}=\frac{C_{A}\times V}{A}$$

(6) $$J_{ss}=\frac{P_{app}}{t}$$

(7) $$ER=\frac{J_{ss}\;formulation}{J_{ss}\;CIL}$$

where P_app_ is the quantity of permeated drug through the membrane (µg/cm^2^), C_A_ is the concentration of CIL in the acceptor chamber (µg/mL), V is the acceptor chamber volume (9 mL), A is the surface of the membrane insert (0.785 cm^2^), J_ss_ is the steady-state flux (µg/cm^2^/h), and t (h) is the duration of the investigation.

#### 4.12.2. BBB-PAMPA Measurement

BBB-PAMPA was used to investigate the CIL permeability (Pe, cm/s) of the prepared formulations and that of the reference (initial CIL solution). The donor plate (Multiscreen™-IP, MAIPN4510, pore size 0.45 μm; Millipore, Merck Ltd., Budapest, Hungary) was pretreated with 5 μL of lipid solution (24 mg of porcine polar brain lipid extract dissolved in 840 μL hexane and 360 μL dodecane [126]). The donor plate was then inserted into the acceptor plate (Multiscreen Acceptor Plate, MSSACCEPTOR; Millipore, Merck Ltd., Budapest, Hungary), which contained 300 μL of PBS solution (pH 7.4). A total of 150 μL of 100 µM reference CIL solution (10 mM CIL solution was prepared in DMSO, then diluted with PBS pH 7.4 to obtain reference donor solution) and freeze-dried formulations were redispersed with PBS (with a nominal concentration of CIL of 3 mg/mL) and transferred to the lipid membrane of the donor plate. Evaporation of solvent from the donor plate was avoided by covering with wet tissue paper and a plate lid. Then, the system was incubated at 37 °C for 4 h (Heidolph Titramax 1000, Heidolph Instruments, Schwabach, Germany), followed by separation of the sandwich system, and CIL concentrations in the donor and acceptor chambers was determined by HPLC. The effective permeability of CIL was calculated using the following equation:

(8) $$P_{e}=-\frac{2.303\cdot V_{A}}{A\left(t-\tau_{ss}\right)}\cdot \log\left(1-\frac{C_{A}\left(t\right)}{S}\right)$$

where P_e_ is the effective permeability coefficient (cm/s), V_A_ is the acceptor phase volume (0.3 cm^3^), A is the filter area (0.24 cm^2^), t is the incubation time (s), τ_SS_ is the time to reach steady state (s), C_A_(t) is the concentration of CIL in the acceptor phase at time point t (mol/cm^3^), and S is the solubility of CIL in the donor phase. The solubility of CIL in donor solutions was determined after preparing a supersaturated solution and centrifuged at 14,000 rpm for 15 min (Eppendorf Centrifuge 5804 R, Thermo Scientific™, Waltham, MA, USA) in Microcon Centrifugal Filter Devices (30,000 Da molecular weight cut-off (MWCO)). Then, the supernatant was diluted 5× in methanol and analyzed using the same RP-HPLC-DAD system. The CIL flux of the formulation was calculated using the following equation:

(9) $$Flux=P_{e}\cdot S$$

For each assay, six parallel measurements were conducted. Data were presented as means ± SD.

### 4.13. Biological Stability Test

The stability of the prepared formulations in the presence of SNES (pH 5.6) and SCSF (pH 7.4) was evaluated in term of Z-average and ZP using a Malvern nanoZS Zetasizer apparatus (Malvern Instruments, Worcestershire, UK). The samples were mixed with SNES and SCSF (55:45% *v*/*v*) and incubated at 35 °C and 37 °C, respectively [127]. The samples were withdrawn at 0, 1, 2, and 3 h, and diluted in the same fluid (1:10 *v*/*v*) using a Fischerbrand P-Series ultrasonic bath (Fischer Scientific Hungary, Budapest, Hungary) for 4 min. The measurements were performed in triplicate. The results were presented as means ± SD.

### 4.14. Statistical Analysis

All results are expressed as means ± SD. GraphPad Prism version 10.12 software (GraphPad Software, San Diego, CA, USA) was used for conducting statistical analysis. One-way analysis of variance (ANOVA) and Tukey’s post hoc test was used to compare the different groups. Data were considered significant when the *p* value < 0.05, asterisks mark * *p* < 0.05, ** *p* < 0.01, *** *p* < 0.001, and **** *p* < 0.0001, and (ns) means a non-significant change.

## Figures and Tables

**Table 1:** Z-average, PDI, and ZP of the prepared formulations. Results are presented as means ± SD (n = 3).

Formulation	Z-Average (nm)	PDI	ZP (mV)
CIL-SPA1	178.97 ± 1.88	0.24 ± 0.02	−61.67 ± 0.21
CIL-SPA2	250.47 ± 3.06	0.20 ± 0.02	−47.40 ± 6.52
CIL-SPA3	336.10 ± 10.15	0.37 ± 0.04	−27.80 ± 0.35
SPA-PG	189.77 ± 4.72	0.36 ± 0.03	−47.23 ± 0.57
SPA-P407	155.73 ± 5.49	0.37 ± 0.04	−27.13 ± 2.40
SPA-CS	273.17 ± 4.37	0.46 ± 0.02	59.80 ± 0.85

**Table 2:** Droplet size distribution of the prepared formulations. Results are presented as means ± SD (n = 3).

Formulation	Dv10 (µm)	Dv50 (µm)	Dv90 (µm)	Span (µm)
CIL-SPA	44.71 ± 5.60	72.38 ± 2.37	131.53 ± 19.35	1.20 ± 0.35
SPA-PG	38.97 ± 1.53	70.16 ± 2.67	139.77 ± 21.87	1.44 ± 0.32
SPA-P407	38.56 ± 1.38	72.98 ± 10.89	127.75 ± 4.88	1.33 ± 0.10
SPA-CS	48.42 ± 1.48	98.58 ± 3.43 **	225.80 ± 7.44 ***	1.80 ± 0.00

**Table 3:** The storage modulus G′, loss modulus G″, and loss factor (tan δ) of the prepared formulations. Results are presented as means ± SD (n = 3).

Formulation	G′ (Pa)	G″ (Pa)	tan δ
CIL-SPA	34.88 ± 18.32	18.48 ± 11.13	0.54 ± 0.13
SPA-PG	795.67 ± 31.83	140.08 ± 16.22	0.18 ± 0.02
SPA-P407	11.20 ± 6.99	4.49 ± 1.55	0.54 ± 0.36
SPA-CS	18.00 ± 10.59	10.71 ± 11.60	0.52 ± 0.30

**Table 4:** The composition of CIL-SPAs.

Formulation	CIL (mg)	Span^®^ 60 (mg)	Tween^®^ 80 (mg)	Oleic Acid (% *w*/*v*)
CIL-SPA1	50	400	100	1
CIL-SPA2	50	300	200	1
CIL-SPA3	50	200	300	1

## Data Availability

Data are available on request to the corresponding authors.

## Supplementary Materials

The following supporting information can be downloaded at: https://www.mdpi.com/article/10.3390/gels11020082/s1, Figure S1: FTIR spectrums of the CIL-SPA formulation in comparison to blank formulation and pure CIL; Figure S2: FTIR spectrums of the SPA-PG formulation in comparison to blank formulation and pure CIL; Figure S3: FTIR spectrums of the SPA-CS formulation in comparison to blank formulation and pure CIL; Figure S4: FTIR spectrums of the SPA-P407 formulation in comparison to blank formulation and pure CIL; Table S1: Comparison of drug release profiles in SNES (pH 5.6) using model dependent approaches; Table S2: Comparison of drug release profiles in PBS (pH 7.4) using model dependent approaches; Table S3: Comparison of drug release profiles in SCSF (pH 7.4) using model dependent approaches.
