# Supplementary material for: Development and Characterization of In Situ Gelling Nasal Cilostazol Spanlastics
Source: Gels. 2025 Jan 22;11(2):82. doi: 10.3390/gels11020082 (PMC11853827; doi:10.3390/gels11020082)
Supplement: Supplementary file 1 [file gels-11-00082-s001.zip › gels-3401380-supplementary.pdf]

Supplementary Materials

Development and characterization of in situ gelling nasal cilostazol spanlastics

Maryana Salamah [1,2], Mária Budai-Szűcs [1], Bence Sipos [1], Balázs Volk [3], Gábor Katona [1,*], György Tibor Balogh [4,5*], Ildikó Csóka [1]

1 1Institute of Pharmaceutical Technology and Regulatory Affairs, Faculty of Pharmacy, University of Szeged, Eötvös Str. 6, H-6720 Szeged, Hungary; salamah.maryana@szte.hu (M.S.); budai-szucs.maria@szte.hu (M.B.S.); sipos.bence@szte.hu (B.S.); csoka.ildiko@szte.hu (I.C.)
2 2Institute of Pharmacodynamics and Biopharmacy, Faculty of Pharmacy, University of Szeged, Eötvös Str. 6, H-6720 Szeged, Hungary; salamah.maryana@szte.hu (M.S.)
3 Directorate of Drug Substance Development, Egis Pharmaceuticals Plc., Keresztúri Str. 30–38, H-1106 Budapest, Hungary; volk.balazs@egis.hu (B.V.)
4 Department of Pharmaceutical Chemistry, Semmelweis University, Hőgyes Endre Str. 9, H-1092 Budapest, Hungary
5 Center for Pharmacology and Drug Research & Development, Semmelweis University, Üllői Str. 26, H-1085 Budapest, Hungary

* Correspondence: katona.gabor@szte.hu (G.K.); balogh.gyorgy.tibor@semmelweis.hu (G.T.B.)

Supplementary Table 1. Comparison of drug release profiles in SNES (pH 5.6) using model dependent approaches.

Model	Model parameters	CIL-SPA	SPA-PG	SPA-P407	SPA-CS
Zero-order	k_0 (min^{-1})	0.935	0.884	0.954	0.890
	R^2	-13.639	-13.241	-10.393	-8.315
	AIC	65.995	64.759	65.654	64.145
	MSC	-2.969	-2.941	-2.718	-2.517
First-order	k_1 (min^{-1})	0.044	0.033	0.044	0.031
	R^2	-3.031	-4.272	-1.971	-2.150
	AIC	56.967	57.803	56.245	56.557
	MSC	-1.679	-1.948	-1.374	-1.433
Higuchi	k_h (min$^{-0.5}$)	9.414	8.832	9.522	8.828
	R^2	-3.231	-3.040	-2.073	-1.380
	AIC	57.307	55.940	56.483	54.594
	MSC	-1.728	-1.682	-1.408	-1.153
Korsmeyer–Peppas	k_{kp} (min^{-n})	38.540	35.544	36.261	31.759
	n	0.146	0.150	0.164	0.179
	R^2	**0.943**	**0.992**	**0.990**	**0.977**
	AIC	29.079	14.304	22.840	23.875
	MSC	2.304	4.265	3.397	3.235

K_0: zero-order release constant; R^2: correlation coefficient; AIC: Akaike Information Criteria; MSC: Model Selection Criterion; K_1: first-order release constant; K_h: Higuchi release constant; K_{kp}: Korsmeyer–Peppas release constant; n: release exponent

Supplementary Table 2. Comparison of drug release profiles in PBS (pH 7.4) using model dependent approaches.

**Model**	**Model parameters**	**CIL-SPA**	**SPA-PG**	**SPA-P407**	**SPA-CS**
Zero-order	k_0 (min^{-1})	1.039	1.038	1.010	1.009
	R^2	0.269	0.465	0.381	0.388
	AIC	51.613	50.099	50.455	50.353
	MSC	-1.121	-0.606	-0.839	-0.831
First-order	k_1 (min^{-1})	0.032	0.029	0.028	0.028
	R^2	0.918	0.966	0.954	0.936
	AIC	38.494	33.525	34.749	36.793
	MSC	1.064	2.156	1.778	1.428
Higuchi	k_h ($min^{-0.5}$)	9.866	9.745	9.530	9.507
	R^2	0.912	0.965	0.950	0.947
	AIC	38.878	33.688	35.298	35.663
	MSC	1.001	2.128	1.6870	1.616
Korsmeyer–Peppas	k_{kp} (min^{-n})	23.089	16.486	18.567	18.564
	n	0.296	0.374	0.340	0.339
	R^2	**0.994**	**0.993**	**0.999**	**0.995**
	AIC	24.699	25.348	13.762	23.547
	MSC	3.3641	3.518	5.276	3.636

K_0: zero-order release constant; R^2: correlation coefficient; AIC: Akaike Information Criteria; MSC: Model Selection Criterion; K_1: first-order release constant; K_h: Higuchi release constant; K_{kp}: Korsmeyer–Peppas release constant; n: release exponent

Supplementary Table 3. Comparison of drug release profiles in SCSF (pH 7.4) using model dependent approaches.

Model	Model parameters	CIL-SPA	SPA-PG	SPA-P407	SPA-CS
Zero-order	k_0 (min^{-1})	1.153	1.180	1.245	1.149
	R^2	-12.344	-3.867	-14.894	-4.691
	AIC	68.115	66.659	70.194	66.370
	MSC	-2.876	-1.868	-3.051	-2.024
First-order	k_1 (min^{-1})	0.104	0.070	0.137	0.067
	R^2	-1.007	0.582	0.533	0.224
	AIC	54.855	49.472	45.499	52.416
	MSC	-0.982	0.587	0.476	-0.031
Higuchi	k_h ($min^{-0.5}$)	11.427	11.521	12.565	11.211
	R^2	-2.909	0.014	-3.678	-0.233
	AIC	59.520	55.479	61.634	55.665
	MSC	-1.649	-0.271	-1.828	-0.495
Korsmeyer–Peppas	k_{kp} (min^{-n})	46.408	33.579	52.507	34.538
	n	0.147	0.233	0.140	0.218
	R^2	**0.888**	**0.985**	**0.945**	**0.987**
	AIC	36.624	27.825	32.448	25.694
	MSC	1.621	3.679	2.340	3.786

K_0: zero-order release constant; R^2: correlation coefficient; AIC: Akaike Information Criteria; MSC: Model Selection Criterion; K_1: first-order release constant; K_h: Higuchi release constant; K_{kp}: Korsmeyer–Peppas release constant; n: release exponent

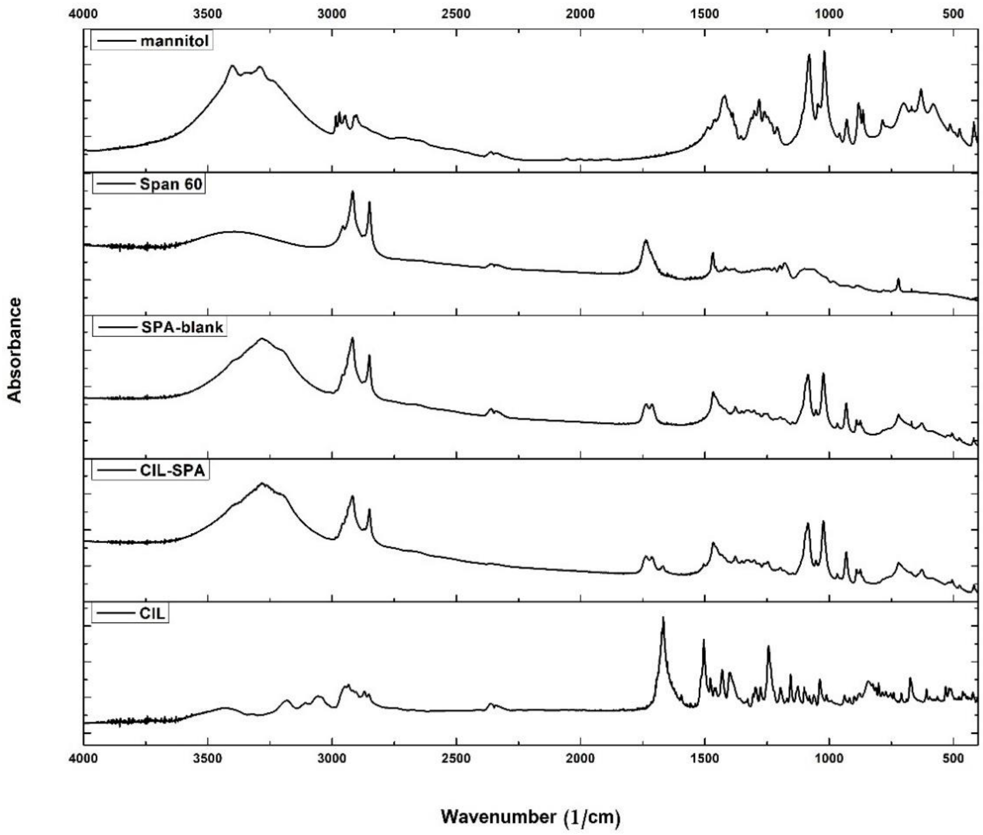

Figure S1: FTIR spectrums of the CIL-SPA formulation in comparison to blank formulation and pure CIL.

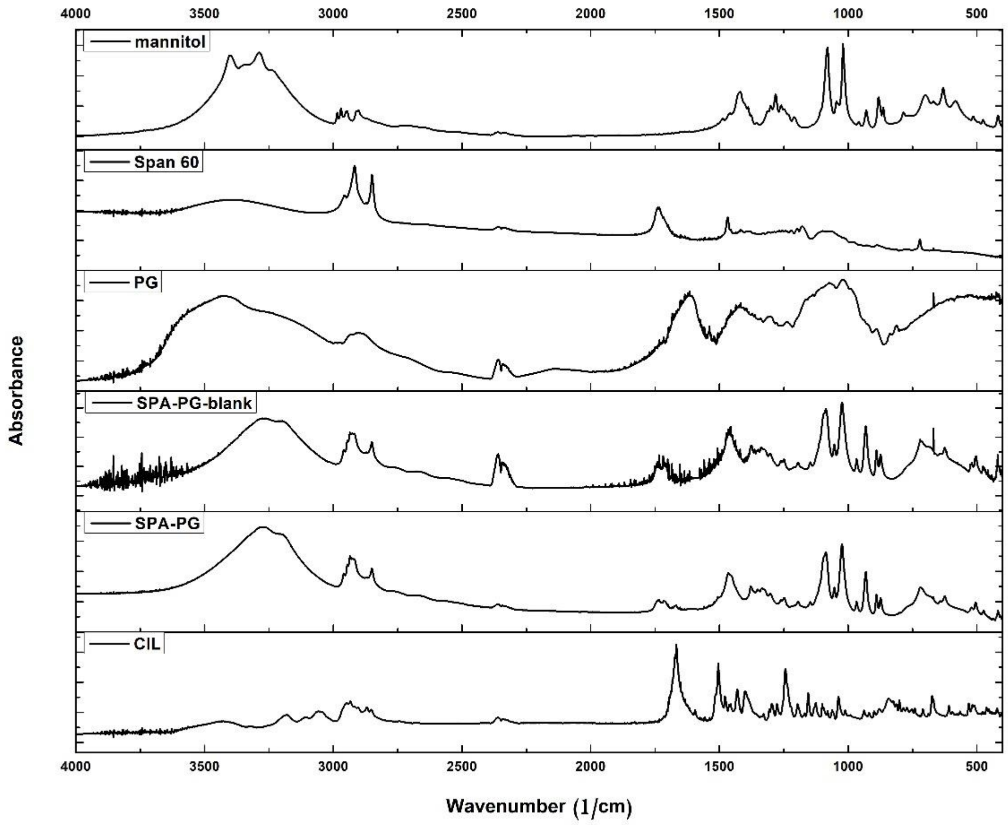

Figure S2: FTIR spectrums of the SPA-PG formulation in comparison to blank formulation and pure CIL.

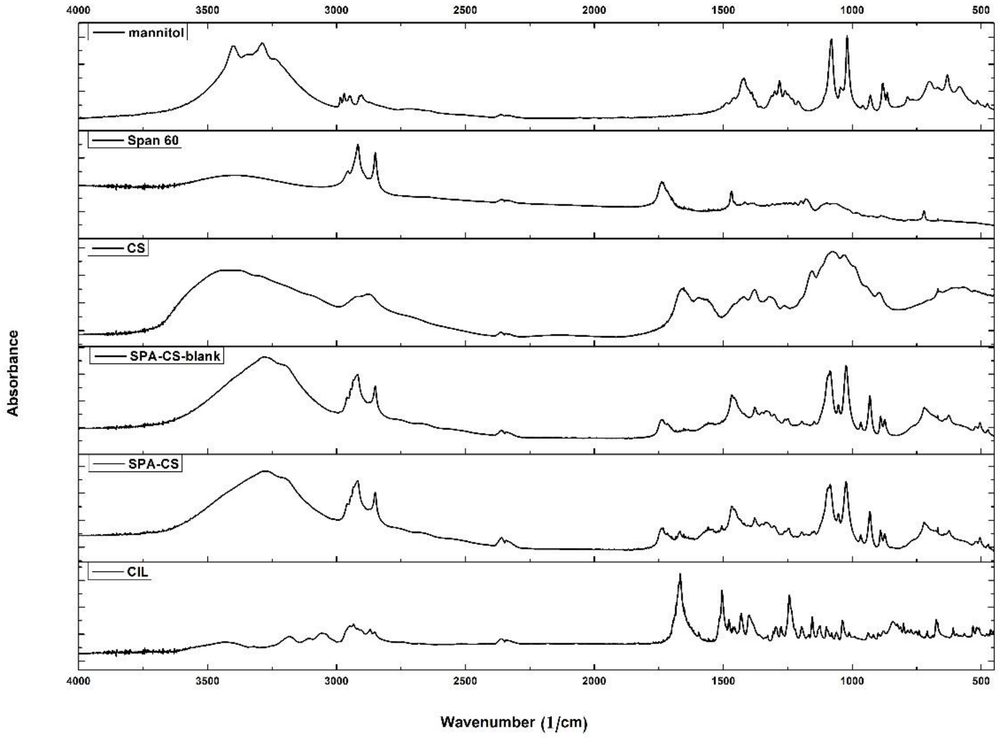

Figure S3: FTIR spectrums of the SPA-CS formulation in comparison to blank formulation and pure CIL.

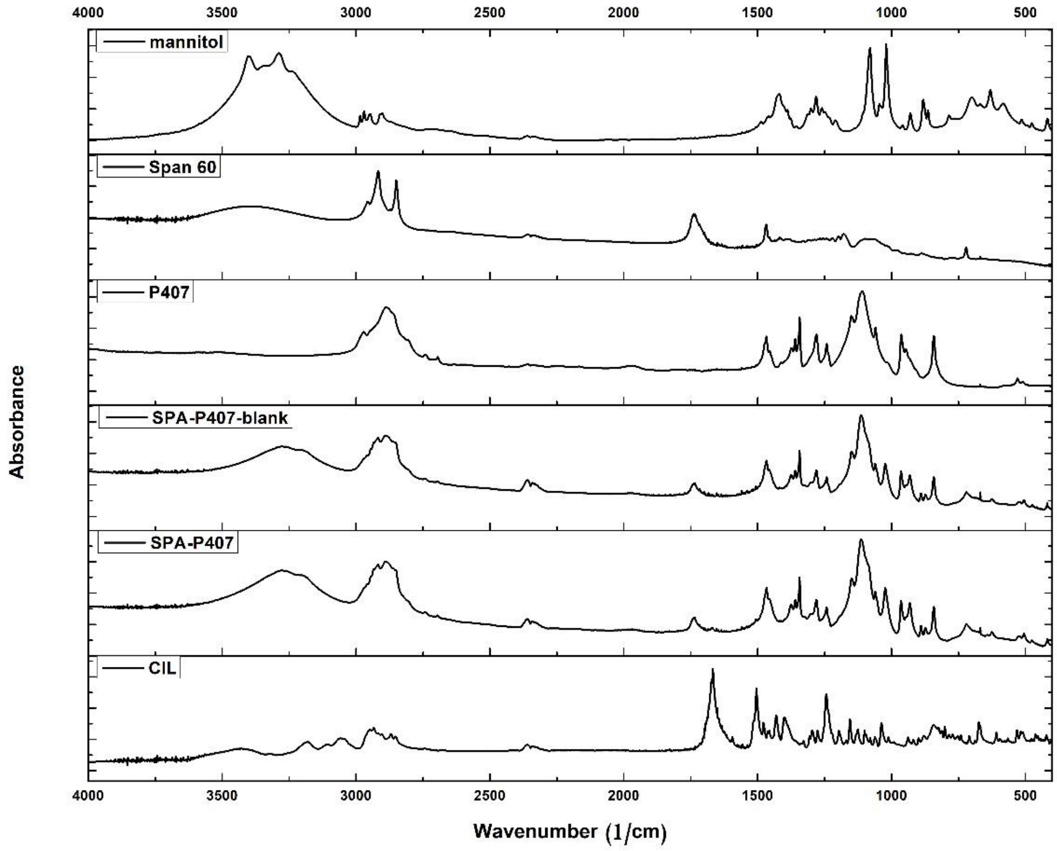

Figure S4: FTIR spectrums of the SPA-P407 formulation in comparison to blank formulation and pure CIL.
